# Early Life Adversity Alters the Developmental Profiles of Addiction-Related Prefrontal Cortex Circuitry 

**DOI:** 10.3390/brainsci3010143

**Published:** 2013-02-04

**Authors:** Heather C. Brenhouse, Jodi L. Lukkes, Susan L. Andersen

**Affiliations:** 1 Department of Psychology, Northeastern University, 360 Huntington Ave, Boston, MA 02115, USA; 2 McLean Hospital, 115 Mill St, Belmont, MA 02478, USA; E-Mails: jlukkes@mclean.harvard.edu (J.L.L.); sandersen@mclean.harvard.edu (S.L.A.)

**Keywords:** maternal separation, prefrontal cortex, dopamine receptors, nucleus accumbens

## Abstract

Early adverse experience is a well-known risk factor for addictive behaviors later in life. Drug addiction typically manifests during adolescence in parallel with the later-developing prefrontal cortex (PFC). While it has been shown that dopaminergic modulation within the PFC is involved in addiction-like behaviors, little is known about how early adversity modulates its development. Here, we report that maternal separation stress (4 h per day between postnatal days 2–20) alters the development of the prelimbic PFC. Immunofluorescence and confocal microscopy revealed differences between maternally-separated and control rats in dopamine D1 and D2 receptor expression during adolescence, and specifically the expression of these receptors on projection neurons. In control animals, D1 and D2 receptors were transiently increased on all glutamatergic projection neurons, as well as specifically on PFC→nucleus accumbens projection neurons (identified with retrograde tracer). Maternal separation exacerbated the adolescent peak in D1 expression and blunted the adolescent peak in D2 expression on projection neurons overall. However, neurons retrogradely traced from the accumbens expressed lower levels of D1 during adolescence after maternal separation, compared to controls. Our findings reveal microcircuitry-specific changes caused by early life adversity that could help explain heightened vulnerability to drug addiction during adolescence.

## 1. Introduction

Early exposure to stress or trauma increases vulnerability to psychiatric disorders that impair motivational processes later in life. Clinically, early childhood adversity is a recognized risk factor for drug addiction [1,2], which typically first emerges during adolescence and early adulthood [3,4,5]. Behavioral changes associated with early adversity are successfully modeled in preclinical studies. Daily repeated removal of rat pups from their mothers (e.g., maternal separation; MS) during the neonatal period is one ethologically-relevant rodent model of early life stress [6]. We [7] and others [8,9,10] have reported that MS can lead to neuronal dysfunction that does not manifest until adolescence, which is consistent with the delayed appearance of several disorders after early life adversity [11]. 

MS reportedly leads to increased alcohol intake [12], greater cocaine self-administration [13], and greater vulnerability to addiction [14,15] in adulthood. However, the mechanistic determinants of these behavioral consequences are not known. The “incentive salience” hypothesis of drug addiction states that dopamine (DA) released in the prefrontal cortex (PFC) and nucleus accumbens (NAc) enhances the perception of stimuli by elevating their salience, or in other words, making them attractive [16]. Attribution of incentive salience to drug-related cues is associated with the expression of DA receptors within the PFC [17]. We previously reported that developmental changes in PFC DA receptors heighten salience attribution to cocaine in adolescent rats [18], however the effects of MS on the PFC DA microcircuitry have not yet been studied. Indeed, effects of early life stress have been largely attributed to changes in hippocampal development and hypothalamic-pituitary-adrenal axis activity [19,20,21,22,23], with few studies focusing on the development of the PFC, and its important role in drug-seeking and addiction [24,25,26].

The PFC is a relatively late-maturing region that is extensively connected with subcortical brain regions and subserves all higher-order cognitive and emotional functions [27]. Chronic stress exposure in adult animals has effects in the PFC such as loss of dendritic spine density and gray matter loss [28,29,30,31]. Within the PFC, the prelimbic region (plPFC) receives dopaminergic (DA) input from the ventral tegmental area and sends dense projections to the nucleus accumbens (NAc), regulating motivational salience attribution and decision-making [32,33] and mediating drug-seeking [34,35,36,37,38,39]. These projections increase linearly with age between the juvenile and adult stages [18]. Amidst the increasing connectivity between regions, adolescence serves as a transition in information processing through the pruning and potential re-focusing of cortical networks [40], which involves functional rearrangement of inhibitory and excitatory control within the PFC [41]. Adolescence is marked by a transient peak in both D1 (D1R) and D2 (D2R) DA receptor densities within the plPFC, followed by pruning in adulthood [42]. Both D1R and D2R expression follow similar developmental profiles in the PFC, with a slightly more dramatic adolescent peak of D1R expression [42]. Consistently, PFC D1R associated second messenger activity rises dramatically between postnatal day 25 (P25; juvenile, pre-adolescent stage) and P40 (adolescence), with a subsequent reduction by P100 (full adulthood) in rats [43].

We previously reported that developmental changes in motivational processing during adolescence are largely mediated by a transient increase of D1R expression specifically on plPFC→NAc projection neurons [18]. D1Rs enhance cortical drive of the NAc, as their role in self-administration [33,44] and reinstatement of conditioned place preference [45,46] suggests. Under addictive states, drug-associated stimuli become selectively important at the expense of weaker stimuli through selective D1R modulation of glutamatergic neurons [33,47]. In contrast, activation of D2R on plPFC projection neurons allows multiple inputs to effectively compete with drug-associated cues, thereby tempering the salience assigned to these cues [33,48]. 

Stress exposure at different developmental time points can differentially affect PFC physiology and function due to its protracted development. For example, adolescent stress exposure decreases overall PFC D2R expression in adulthood [49]. To date, little is known about whether altered development initiated early in life can impact the normal developmental trajectory of the plPFC during its active transition to maturity. However, recent reports that MS increases dendritic growth and spine density in the adult PFC and NAc [50] suggest that there are important MS effects on PFC circuitry. Here, we aimed to determine whether MS disrupts the adolescent peak in plPFC DA receptor expression that mediates heightened adolescent addiction-like behaviors by examining their expression on glutamatergic projection neurons. 

## 2. Results and Discussion

### 2.1. Experiment 1: Effects of Early Life Stress on Overall DA Receptors and Glutamatergic Projection Neurons in the plPFC across Development

**Figure 1:**
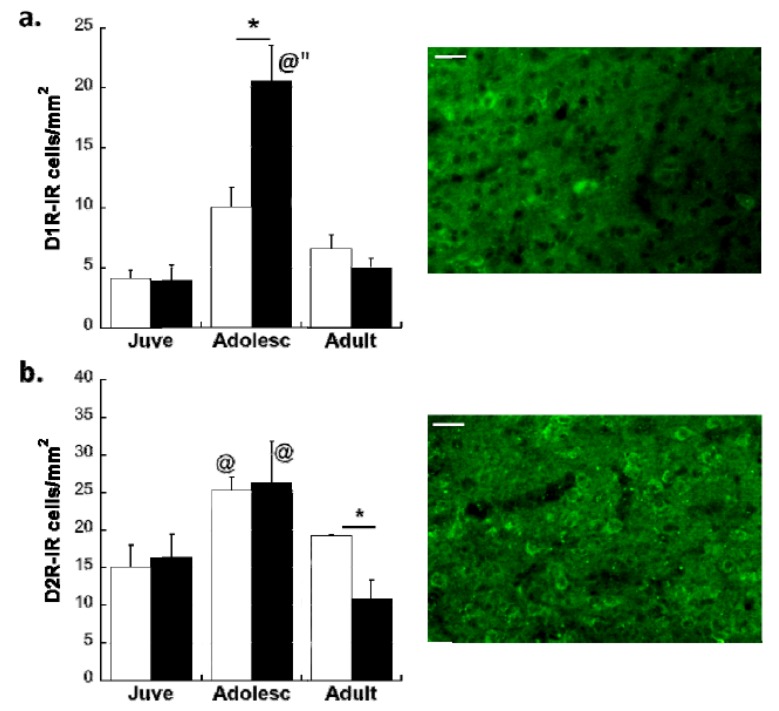
(**a**) Effects of MS on overall D1R density in the plPFC. White bars: CON; Black bars: MS. Averages ± SEM are presented. *n* = 5–6. * *p* < 0.05 difference between stress groups, ^@^* p* < 0.05 difference from juveniles using *post-hoc* Bonferroni *t*-tests after Age × Stress Group interaction was found. (**b**) Effects of MS on overall D2R density in the plPFC. * *p* < 0.05 difference between groups using independent *t*-tests (Note: No Age × Stress Group interaction was found using 2-way ANOVA). ^@^* p* < 0.05 difference from juveniles using independent *t*-tests. Main effect of Age: *F*(2,25) = 2.015, *p* = 0.031. White bars: Con; Black bars: MS. Averages ± SEM are presented. *n* = 5–6. Representative photomicrographs from adolescent Con subjects are shown with each graph. Scale bar: 20 µm.

Neither MS exposure nor age significantly affected the volume of the plPFC (MS P25: 3.8 ± 0.06 mm^3^; P40: 4.1 ± 0.4 mm^3^; P100: 4.7 ± 0.4) and CON groups (P25: 4.2 ± 0.26 mm^3^; P40: 4.4 ± 0.39 mm^3^; P100: 5.6 ± 0.4). Consistent with previous observations in the PFC [40], overall D1R expression (main effect of Age: *F*(2,23) = 20.44, *p* < 0.001; see Figure 1a) and D2R expression (main effect of Age: *F*(2,25) = 2.015, *p* = 0.031; see Figure 1b) in the plPFC changed throughout development and reached their highest level during adolescence in CON animals before a decrease in adulthood. MS caused an increase of D1R that was 2-times the adolescent increase in D1R (Age × Stress Group interaction: *F*(2,23) = 7.308; *p* = 0.003). However, MS did not significantly affect overall expression of D2R in the adolescent plPFC. Despite a lack of Age × Stress Group interaction, *post-hoc* comparisons did reveal an effect of MS on D2R in adulthood, with lower D2R levels in MS adults (*t*(8) = 3.11; *p* = 0.014). It was therefore important to determine if MS alters DA receptor expression on functionally specific neuronal types. While D1R do not typically peak on inhibitory interneurons, MS may cause an abnormal increase of D1R on plPFC interneurons during adolescence. However, preliminary counts performed in our laboratory do not suggest that this is the case (data not shown). Therefore, effects of MS on glutamatergic PFC projection neurons are shown in the experiments below. 

#### Effects of Age and MS on D1R and D2R Dopamine Expression Distribution on All Glutamatergic Projection Neurons in the plPFC

**Figure 2:**
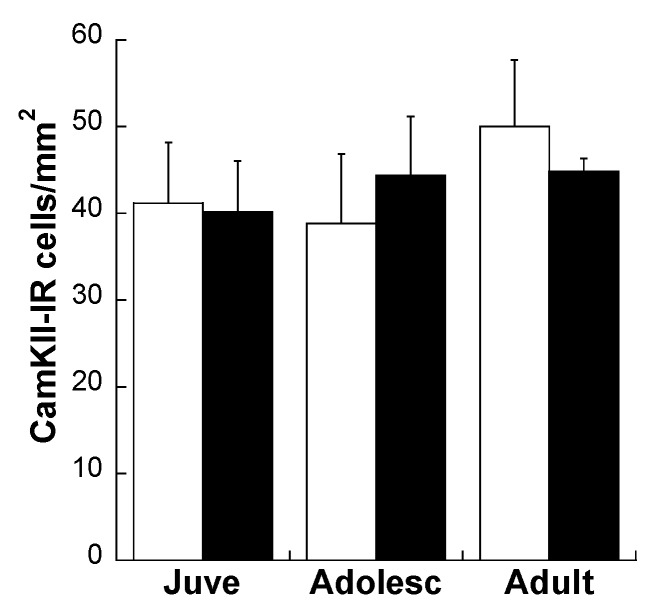
Density of CamKIIa-immunoreactive (IR) cells per mm^2^ in the plPFC of CON (white bars) and MS (black bars) rats at three ages. Averages ± SEM are represented.

Immunofluorescent labeling of CamKIIa revealed no effect of either age or MS on overall glutamatergic projection neurons in the plPFC (Figure 2; *p* = 0.908). 

**Figure 3:**
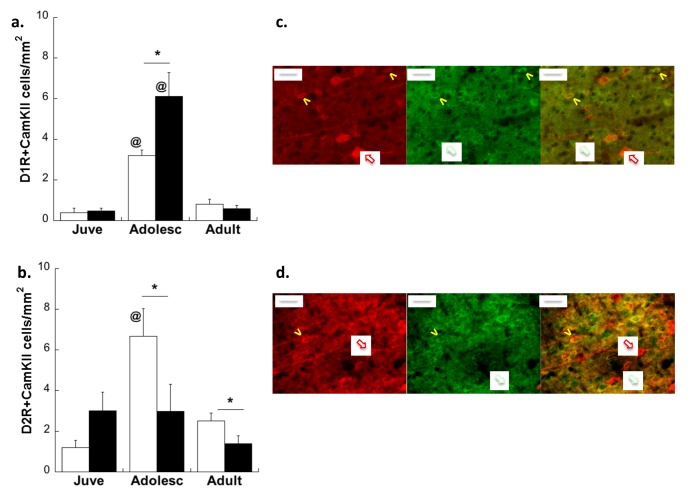
D1R (**a**,**c**) and D2R (**b**,**d**) expression changes on CamKIIa-IR glutamatergic neurons after MS in the plPFC. * *p* < 0.05 difference between groups using Bonferroni *post-hoc t*-tests after 2-way ANOVA. ^@^* p* < 0.05 difference from juveniles. White bars: CON; Black bars: MS. Averages ± SEM are presented. *n* = 5–6. Representative photomicrographs of individual channels and overlays from control adolescents taken at 40× are shown in (**c**) and (**d**). Red-outlined white arrows point to CamKIIa neurons, green-outlined white arrows point to D1R (**c**) or D2R (**d**) neurons, and yellow pointers point to co-localized neurons. Scale bar: 20 µm.

As expected, the adolescent peak of D1R (main effect of Age: *F*(2,24) = 32.01; *p* < 0.0001) and D2R (main effect of Age: *F*(2,24) =29.83; *p* = 0.003) was found to occur on these projection neurons (Figure 3). However, MS disrupted the normal developmental trajectory of DA receptor expression on plPFC glutamatergic neurons. Animals subjected to MS displayed a 79% higher peak of D1R in adolescence compared to CON (Age × Stress Group interaction: *F*(2,24) = 4.64; *p* = 0.02; Figure 3a). *Post-hoc* comparisons revealed more D1R + CamKIIa neurons in MS adolescents compared to CON adolescents (*t*(10) = −2.37; *p* = 0.039). In contrast, adolescent rats subjected to MS displayed a 60% reduction in expression of D2R on plPFC projection neurons compared to their CON counterparts (Age × Stress Group interaction: *F*(2,24) = 4.78; *p* = 0.018; Figure 3b). *Post-hoc* analysis of D2R + CamKIIa showed lower colocalized neurons in MS adolescents compared to CON adolescents (*t*(10) = 2.38; *p* = 0.04). Effects of MS on D2R endured into adulthood, as MS adults also displayed lower D2R expression on CamKIIa-IR cells (*t*(8) = 5.75; *p* < 0.001).

### 2.2. Experiment 2: Effects of Early Life Stress on D1R and D2R Distribution Specifically on plPFC→NAc Neurons across Development

**Figure 4:**
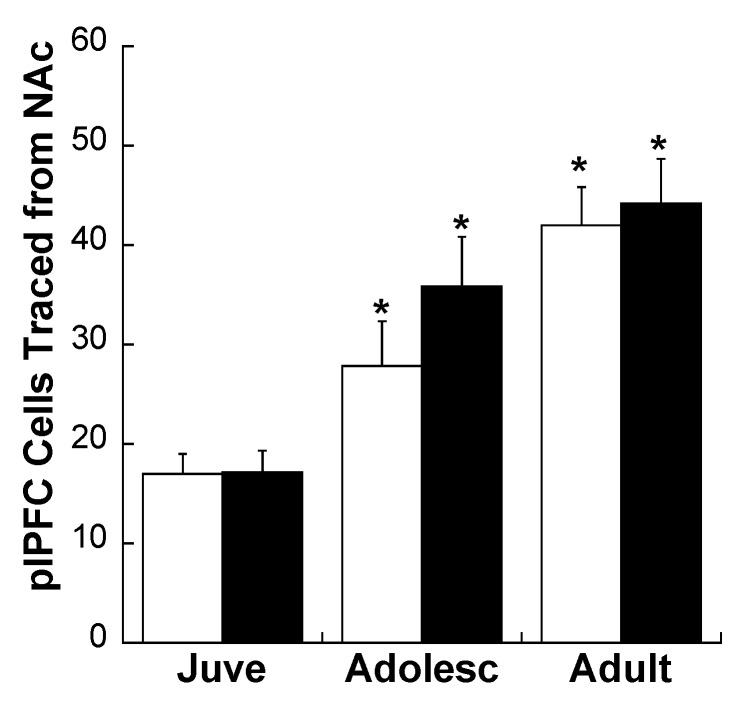
Density of traced cells per mm^2^ in the plPFC of CON (white bars) and MS (black bars) rats at three ages. * *p* < 0.05 difference from juveniles within the same stress group. Averages ± SEM are presented.

Consistent with our previous report [18], plPFC projections to the NAc core increased with age (see Figure 4; main effect of Age: *F*(2,17) = 5.212; *p* = 0.017). There was no Age × Stress Group interaction (*p* = 0.182), nor a main effect of Stress Group (main effect of Stress Group: *p* = 0.248). 

**Figure 5:**
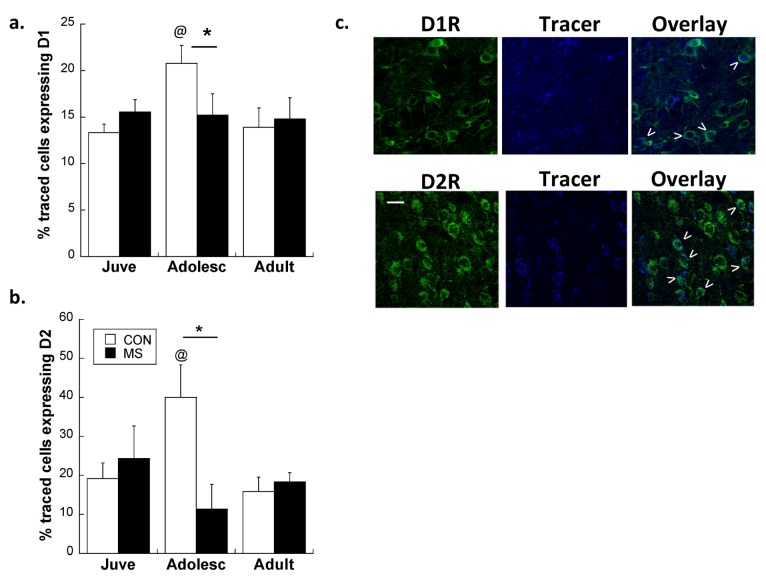
Peak D1R (**a**) and D2R (**b**) expression on PFC→NAc projections in adolescence is absent after MS. * *p* < 0.05 difference between stress groups using Bonferroni *post-hoc t*-tests after 2-way ANOVA. ^@^* p* < 0.05 difference from juveniles. White bars: CON; Black bars: MS. Averages ± SEM are presented. Representative photomicrographs from CON adolescent animals displaying receptor-ir, retrograde tracer, and colocalization (arrows) are shown in (**c**). Bar: 20 µm. Images cropped and enlarged for clarity. *n* = 6 for all treatment groups.

A focused study of projection neurons bound for the NAc specifically revealed important distinctions in how MS affects plPFC microcircuitry. Confocal analysis of traced neurons co-expressing D1R and D2R revealed an interaction between group and age for both D1R and D2R. Notably, while MS was found to exacerbate the adolescent increase of D1R on all glutamatergic neurons, the peak of D1R on plPFC→NAc neurons was decreased by 27% in MS adolescents compared to CON adolescents (Age × Stress Group interaction: *F*(2,22) = 6.4; *p* = 0.006; Figure 5a). *Post-hoc* comparisons revealed that this interaction was driven by differences between MS and CON adolescents (*t*(9) = 2.27; *p* = 0.05). While D1R was differentially affected on plPFC→NAc neurons compared to the overall population of glutamate neurons, MS had consistent effects on D2R across all projection neurons, with MS adolescents displaying fewer D2R on plPFC→NAc neurons during adolescence compared to CON (Age × Stress Group interaction: *F*(2,22) = 8.1; *p* = 0.002; Figure 5b). *Post-hoc* analyses of D2R-ir traced neurons revealed that MS adolescents displayed significantly less D2R expression on plPFC→NAc cells than CON adolescents (*t*(9) = 3.79; *p* = 0.004). In other words, traced neurons in CON adolescents expressed significantly more D1R and D2R than juveniles and adults (*p* < 0.05 for both receptors), but MS adolescents did not display a similar peak for either receptor. 

We report here that MS disrupts the development of the plPFC microcircuitry that mediates motivational salience and drug-seeking behaviors. Specifically, early traumatic experience—modeled here with MS—leads to a disruption in a DA receptor profile that provides important inhibitory/excitatory balance during adolescence. Typically, adolescent rats demonstrate a transient increase in both D1R and D2R expression on glutamatergic plPFC projection neurons (current report and see [18]). The increased D1R and D2R on projection neurons act to focus motivated behavior towards important stimuli—including drug-related stimuli—during adolescence [18], allowing maturation into a self-directed adult. We previously showed that a transient increase of D1R on excitatory plPFC projections (but not inhibitory interneurons) during adolescence enables the acquisition of conditioned place preference for cocaine during adolescence [18]. Adolescence is a transition in information processing through the pruning and potential re-focusing of cortical networks [40], which involves functional rearrangement of inhibitory and excitatory control within the PFC [18,41]. D1R contribute to excitation of both interneurons and excitatory projection neurons in the PFC, and are necessary for addiction-related behaviors such as reinstatement of drug-seeking [51] and conditioned place-preference [18,45]. D2R, however, attenuate excitability of PFC projection neurons, tempering D1-mediated activation [52] by fine-tuning information flow to subcortical structures [53,54]. Here, we observed a transient increase in both D1R and D2R expression on projection neurons during adolescence. MS heightens the increase of D1R on glutamatergic neurons during adolescence while also diminishing the expression of D2R, which may shift the control of PFC output to D1R activity. Since D1R have an excitatory effect on PFC output, increased D1R drive in the PFC may increase excitability of subcortical limbic nuclei in MS animals. 

The overexpression of D1R that we observed during adolescence is consistent with the peak in DA afferent density to pyramidal (but not non-pyramidal) cells in the PFC around puberty [55]. After MS, increased D1R in the PFC is consistent with previously observed spine density increases after early life adversity [50], since PFC D1R are enriched in dendritic spines [56]. In contrast to the increase of D1R on glutamatergic projection neurons overall, we observed that MS prevents the typical adolescent peak of D1R on the subset of these neurons that project to the NAc. This was a surprising finding, since D1R in the plPFC→NAc tract is instrumental in controlling reward processing and motivational salience. For example, repeated exposure to cocaine in adult animals increases D1-like activity selectively on these NAc-targeting projection pyramidal neurons and enhances craving [33]. It is possible that decreased D1R in plPFC→NAc core neurons could lead to an abnormally low baseline that lends itself to more dramatic cocaine-induced increases in D1R in MS adolescents. Moreover, there are several other termination sites of the plPFC that also play important roles in drug seeking and motivation, making D1R expression on non-accumbal projection neurons equally relevant. For example, the basolateral amygdala (BLA) receives excitatory input from the plPFC and in turn projects heavily onto the NAc core [57]. Disruption in PFC-BLA communication has been shown to increase risky behaviors and decrease sensitivity to negative feedback—two critical components of drug addiction and other adolescent disorders [58,59]. Taken together, these data demonstrate the importance of distinguishing not only cell type, but also cell connectivity, when analyzing receptor changes in the PFC.

After MS, lower levels of D2R on excitatory projection neurons could contribute to a D1-dominant modulation of the PFC, which has been postulated as a key mechanism of drug-seeking [33]. Reduced PFC D2R is also attributed to long-term cocaine exposure, and is proposed to be responsible for the cognitive deficits and compulsive drug taking seen in addicts [60]. Therefore, MS may also precipitate a vulnerability to compulsive drug-taking via altered D2R expression profiles in the PFC. Interestingly, MS effects on D2R endured into adulthood, which speaks to longer lasting changes that may cause risk for addiction even if an individual evades vulnerability during adolescence.

## 3. Experimental Section

### 3.1. Subjects

Pregnant female multiparous Sprague-Dawley rats (250–275 g) were obtained from Charles River Laboratories (Wilmington, MA) on day 13 of gestation. The day of birth was designated as postnatal day 0 (P0). One day after birth, litters were culled to 10 pups (7 males and 3 females), and litters were randomly assigned to either a maternal separation group (MS Group) or animal facility reared control group (CON Group). Pups in the MS Group were isolated for 4 h per day between P2 and P20, and kept at thermoneutral temperature. This procedure is similar to procedures used previously by this laboratory [7,19,61] and others [62]. Pups in the CON Group were not disturbed after day 2, except for routine weekly changes in cage bedding, during which all pups were weighed. Rats were housed with food and water available *ad libitum* in constant temperature and humidity conditions on a 12-h light/dark cycle (light period 07:00–19:00). Rats were weaned on P21–P22, and group-housed in same-sex caging until the time of surgery. 

These experiments were conducted in accordance with the 1996 Guide for the Care and Use of Laboratory Animals (NIH), and were approved by the Institutional Animal Care and Use Committee at McLean Hospital.

### 3.2. Experiment 1: Effects of Early Life Stress on DA Receptors and Glutamatergic Projection Neurons in the plPFC across Development

Fourteen male juvenile rats (which were P25 when brains were harvested), *n* = 14 male adolescent rats (which were P39 on surgery day and P44 when brains were harvested) and *n* = 14 male adult rats (which were P100 when brains were harvested) were separated into two groups with differing early experience. Therefore, six experimental groups (*n* = 7) were analyzed: Juvenile CON, Juvenile MS, Adolescent CON, Adolescent MS, Adult CON, and Adult MS. In all studies, only one rat per litter was used per age in order to avoid litter effects [63] One-two animals per group were omitted from final analyses due to sub-optimal immunohistochemical staining.

#### Double-Label Immunofluorescence

Tissue was processed with standard immunohistochemical methods [18]. Briefly, animals were intracardially perfused with ice-cold PBS followed by 4% paraformaldehyde, and brains were sliced in 40 µm sections on a freezing microtome. Sections were incubated overnight in mouse anti-Calmodulin-Kinase IIa IgG (CamKIIa; 1:1000; EMD Millipore, Bellerica MA; to label glutamatergic neurons) and either rat anti-D1 IgG (1:250; Sigma) or rabbit anti-D2R IgG (1:400; Millipore), washed, and incubated for 60 min with anti-rat Alexa 563-coupled IgG (1:400; Molecular Probes, Eugene, OR) and anti-rabbit Alexa 488-coupled IgG (1:400; Molecular Probes). Sections stained for D1R were immediately adjacent to sections stained for D2R. Sections were then washed and mounted on slides using FluoroGel mounting medium (Fisher Scientific, Pittsburgh PA). Stereo Investigator Image Analysis System (MBF Bioscience, Williston VT) was used to estimate the density of CamKIIa-, D1R-, and D2R-positive cells. The plPFC in four serial coronal sections (intersection interval 240 µm) per animal were analyzed [64] for either CamKIIa + D1R or CamKIIa + D2R. This encompassed an area from 2.2 to 1.1 mm from Bregma. In each section, the entire plPFC was outlined at 4× magnification and the total number of IR cells was measured at 20× exclusively within the outlined area. CamKIIa was visualized using a red filter channel, while D1R and D2R were visualized using a green filter channel. Double-labeled cells were confirmed using an overlay of images from two filters for each field of view. Investigators were strictly blinded to the conditions for all analyses. Tracings of the plPFC boundaries were used for calculation of the area (*a*) in each section. The density of IR cells for each cell type (cells/mm^2^) was based on the total number of IR cells divided by Σ*a* for each subject (the sum of areas obtained from all outlined regions). Volume of the plPFC was calculated according to the Cavalieri principle [65] as *v* = *z* × *i* × Σ*a*, where *z* is the thickness of the section (40 µm) and *i* is the section interval (24; *i.e.*, number of serial sections between each section and the following one within a compartment). Group differences were determined by 2-way (Age × Stress Group) ANOVA. Bonferroni’s *post-hoc t*-tests compared group means after interactions were found.

### 3.3. Experiment 2: Effects of Early Life Stress on D1R and D2R Distribution Specifically on plPFC→NAc Neurons across Development

In order to determine whether changes in DA receptor distribution included, or were specific to, the plPFC→NAc tract, a separate cohort of *n* = 12 male juvenile rats, *n* = 12 male adolescent rats, and *n* = 12 male adult rats were used. Within each age, half of subjects were from the MS Group and half were from the CON Group as described above. Therefore, six groups of *n* = 6 rats were injected with retrograde tracer into the NAc and analyzed for expression of D1R and D2R.

#### 3.3.1. Retrograde Tracer Microinjections

**Figure 6:**
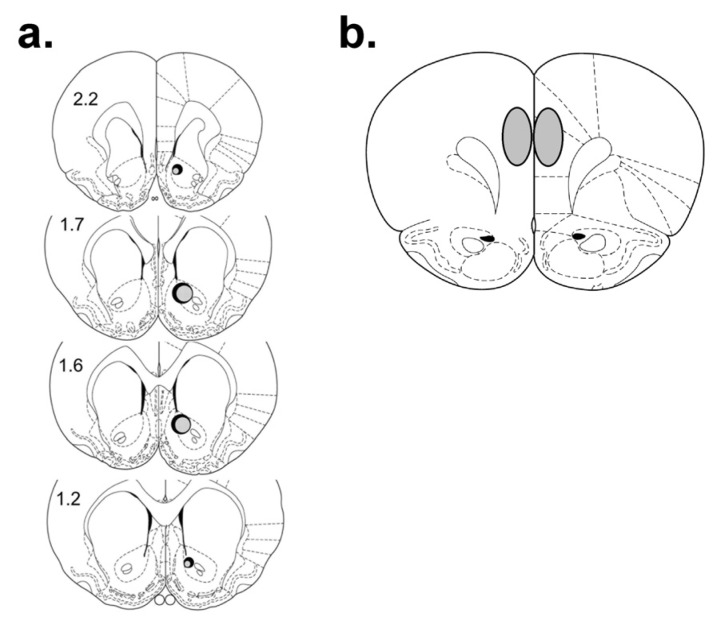
Histological analysis of intra-NAc fluosphere injection sites (**a**) and the region of plPFC that comprised the largest density of retrogradely-traced neurons, and was therefore analyzed (**b**). Numerals indicate mm from bregma. The largest (solid) and smallest (shaded) bolus sizes of all animals included in analysis are indicated. All injection sites were within ±10% in size.

In order to define the discrete region of plPFC where projections to the NAc reside, retrograde tracer was injected into the NAc core [18]. Rats were anesthetized with a ketamine/xylazine mixture and a Hamilton syringe was stereotaxically lowered into the NAc core. Coordinates for juveniles were AP: +2.1, ML: +1.3, DV: −6.5 and coordinates for adolescents were AP: +1.8, ML: +1.2, DV: −6.8 [64]. Coordinates for adults were AP: +1.6; ML: +1.2, DV: −6.8 [66]. One micrometer of 660λ fluospheres (Molecular Probes) was injected over 2 min, after which the needle remained in the brain for an additional minute to ensure diffusion into the surrounding tissue, based on our previous methodology [18]. Fluospheres were used because fibers of passage are not affected such that only direct projections are stained [67]. Fluosphere bolus spread and intensity was measured using Leica confocal software (Leica Microsystems, Heidelberg, Germany) to verify that a consistent amount of tracer was introduced into all animals, as previously reported ([18]; Figure 6).

#### 3.3.2. D1 and D2 Fluorescence Immunohistochemistry

Five to seven days following retrograde tracer microinjections, tissue was processed for D1- and D2-IR as described above. Sections were washed, mounted on slides, and imaged with confocal microscopy.

#### 3.3.3. Confocal Microscopy and Analysis

High magnification digitized images of neurons showing retrograde tracer were made with a 40× oil immersion lens and confocal microscopy (Leica). A *z*-series stack of images was acquired using 2 μm-step intervals for 7–10 consecutive focal planes. Regions of interest (ROI) were chosen within the plPFC based on prominence of retrograde fluosphere-labeled cells as identified by scanning at 10× (Figure 6). Within each ROI, *z*-series stacks were generated for each individual cell with the FITC [for D1R- and D2R-like IR], and Cy5 filters (to view retrograde fluospheres), respectively to guard against false-positives. Three ROIs were generated per section. Within each region, individual D1- or D2-labeled cells were counted, selected and measured for colocalization across focal planes. Due to the age-dependent changes in tracer-labeled neurons, the percentages of total tracer-labeled cells that were colocalized with D1R or D2R were recorded, and these values were compared between ages and stress group using 2-way ANOVA with Tukey’s LSD *post-hoc* analysis, unless otherwise noted. 

## 4. Conclusions

Here we report that early life adversity leads to discrete changes in plPFC DA receptor expression profiles during adolescence, thereby upsetting an already delicate regulation of motivational behavior during a tumultuous stage of life. Adolescence is an important transitional stage that comprises many of the behaviors that characterize substance use [68,69], placing adolescents at increased risk for drug abuse and addictions [70]. Our findings corroborate with reports that early adversity is reported to account for one half to two-third of serious problems with drug use later in life [71]. Specifically, we found that early adversity leads to exacerbation of the adolescent increase of D1R that is already thought to confer vulnerability to drug-taking and abuse. In conjunction, we found a blunted increase of D2R during adolescence, which may normally serve to temper the D1R-induced hyper-focus on strong rewards. Although growing evidence implicates cortical maturation in the high vulnerability to addiction in adolescence [40], little has been known about early environmental influences on the receptor systems that mediate incentive salience [72]. Consideration of the nature and timing of such neuroadaptations may provide a basis on which to identify and treat an at-risk population.
